# Enhancing Tuberculosis Treatment Adherence: Evaluating the Efficacy of the Support for Treatment Adherence and Medication Protocols (STAMP) Device for Automatic Dispensing and Real-Time Medication Monitoring

**DOI:** 10.7759/cureus.69611

**Published:** 2024-09-17

**Authors:** Simon Chandramohan Jason Charles, Krishna Anusha, Krishna Mahesh, Ramachandran Ramasubramanian, Perumal Kaliraj, Vimalraj Selvaraj

**Affiliations:** 1 Biostatistics, Sundaram Medical Devices Private Limited, Mylapore, IND; 2 Research, Sundaram Medical Devices Private Limited, Mylapore, IND; 3 Biotechnology, Anna University, Chennai, IND; 4 Applied Mechanics and Biomedical Engineering, Indian Institute of Technology Madras, Chennai, IND

**Keywords:** automatic medicine dispenser, medication adherence strategies, real-time monitoring, tablet, tuberculosis

## Abstract

Tuberculosis (TB) remains a significant global health challenge, necessitating strict adherence to medication for successful treatment and prevention of drug resistance. Adherence to a regular medication regimen is crucial in TB management, yet achieving high adherence rates among patients proves challenging due to various factors including forgetfulness, complexity of treatment schedules, and socioeconomic barriers. This study explores the potential of an automatic medicine dispenser (AMD) anchored system to improve medication adherence among TB patients and evaluates its impact through monitoring and feedback mechanisms. The AMD integrates advanced technology to dispense medications at scheduled times, thereby reducing reliance on patient memory and manual adherence tracking. This device is complemented by a monitoring system where healthcare mentors oversee adherence data in real-time via a web-linked dashboard. Such real-time monitoring enables mentors to promptly intervene in cases of non-adherence, offering personalized support and guidance tailored to individual patient needs. The study employs a mixed-methods approach, combining quantitative analysis of adherence rates derived from the AMD anchored system with qualitative data from patient surveys. These surveys gather insights into patient experiences and perceptions regarding the use of the AMD anchored system, including feedback on the accuracy of medication intake recorded by the device. Data obtained from the surveys are analyzed alongside adherence metrics from the dashboard to elucidate factors influencing adherence behavior and the device's effectiveness in fostering sustained treatment adherence. Preliminary findings indicate promising outcomes associated with the AMD anchored system intervention. High rates of adherence are observed among patients utilizing the device, attributed to the device's automated dispensing mechanism and the supportive role of healthcare mentors. Challenges such as technical malfunctions and patient acceptance are also identified, prompting continuous refinement of the AMD and mentorship strategies. In conclusion, the integration of an AMD coupled with real-time monitoring through a web-linked dashboard represents a significant advancement in TB treatment adherence management. Future research should focus on the scalability and sustainability of this technology-augmented, holistic approach across diverse healthcare settings to maximize its impact on global TB management strategies.

## Introduction

Tuberculosis (TB) remains a significant global health concern, posing challenges to healthcare systems worldwide. According to the WHO, TB is one of the top 10 causes of death globally and the leading cause from a single infectious agent, ranking above HIV/AIDS. In 2020, an estimated 10 million people fell ill with TB worldwide, with approximately 1.5 million succumbing to the disease. The burden is particularly heavy in low- and middle-income countries, where factors such as poverty, overcrowding, and limited access to healthcare exacerbate transmission rates and hinder effective treatment [1,2]. In India, TB presents a substantial public health challenge due to its high burden of cases. India accounts for about a quarter of the global TB cases, with an estimated 2.64 million people developing TB in 2020. Despite efforts to control the disease, challenges such as diagnostic delays, drug resistance, and barriers to healthcare access persist. The Indian government has implemented extensive TB control programs, including the Revised National Tuberculosis Control Program (RNTCP), to enhance case detection, treatment adherence, and overall management. Innovative approaches, including the adoption of digital technologies and community engagement strategies, are being explored to accelerate progress towards TB elimination goals set by the WHO [3].

TB is notorious for its persistence and severity, exacerbated by the necessity for strict adherence to a prolonged medication regimen. Non-adherence can lead to treatment failure, drug resistance, and higher mortality rates, highlighting the critical importance of consistent medication intake [4]. Automated medication dispensing (AMD) systems have emerged as a promising solution to enhance adherence among TB patients. These systems not only automate the dispensing of medications but also integrate monitoring and support mechanisms to ensure patients adhere to their prescribed treatment plans. By leveraging technology, AMDs provide real-time tracking via online dashboards, facilitate regular reminders, and enable timely interventions through mentor calls when necessary [5]. This paper presents the implementation and evaluation of an AMD system designed specifically for TB patients. We assess its impact on treatment adherence and health outcomes compared to conventional methods. Additionally, a qualitative and statistical analysis, including survey data, examines patient experiences and efficacy indicators. Preliminary results indicate promising improvements over traditional approaches, suggesting that AMD systems could revolutionize TB treatment management [6], [7]. Through this study, we aim to contribute valuable insights into the effectiveness of AMD systems in enhancing medication adherence and ultimately improving health outcomes for TB patients worldwide. The AMD used in Support for Treatment Adherence and Medication Protocols (STAMP) represents a cutting-edge electronic medication dispenser designed to enhance adherence in healthcare settings, particularly for patients undergoing treatment for TB. Comprising three distinct models, STAMP UltraPortable, STAMP Camouflaged, and STAMP UltraCapacity, the device caters to diverse patient needs with varying requirements for portability, privacy, and medication capacity. Functionally, STAMP operates with simplicity and efficacy: at prescribed medication times, it emits audible reminders and delivers precise doses directly into the patient's hand upon activation. This action triggers automatic updates to a secure, remote database via robust SMS protocols, ensuring real-time recording of medication administration. In cases of missed doses, the system initiates a progressive notification protocol, beginning with SMS alerts and culminating in automated voice calls to patients and SMS to patient-designated caregivers or healthcare workers (HCWs) for timely intervention [7]. Manufactured entirely in India, STAMP underscores its commitment to local sourcing and production, contributing to regional economic development and sustainability. Technical operations involve seamless integration with existing TB treatment workflows, facilitating streamlined initiation, refill processes, and troubleshooting procedures under the guidance of HCWs or pharmacists. This study explores STAMP's technological framework, deployment strategies, and the significant impact observed in healthcare outcomes, thereby advocating for its integration as a pivotal tool in global TB management strategies. The analysis of data from the STAMP dashboard, with the overarching goal of improving treatment outcomes, serves a dual purpose: first, to assess medication adherence and device efficacy among TB patients, and second, to identify reasons for non-adherence through follow-up details logged in the system. The dashboard aggregates real-time data on medication dispensation events, providing quantitative measures of adherence rates and timeliness of medication intake. This data is crucial for evaluating the operational effectiveness of the STAMP device in supporting treatment adherence. Additionally, the dashboard facilitates a granular analysis of non-adherence factors by tracking the escalation of notifications sent to patients and caregivers upon missed doses. Common reasons for non-adherence, such as forgetfulness or logistical challenges, can be identified through this process, informing targeted interventions to improve patient compliance. To gather sensitive information regarding actual medication consumption by patients using STAMP, a randomized response technique (RRT) is employed. RRT ensures confidentiality by introducing random noise into responses from selected patients, thereby safeguarding individual privacy while enabling accurate estimation of medication consumption rates. This methodological approach is essential in maintaining ethical standards and patient confidentiality while conducting detailed analyses of treatment adherence and device efficacy in clinical settings [8]. Altogether, leveraging data analytics from the STAMP dashboard, coupled with RRT for sensitive information, provides comprehensive insights into medication adherence behaviors and the operational performance of the device. These insights are instrumental in optimizing TB treatment protocols and enhancing patient outcomes within healthcare systems.

## Materials and methods

Ethical clearance

In accordance with ethical guidelines, appropriate clearance was obtained from the Institutional Ethics Committee (IEC) prior to conducting surveys involving TB patients using the STAMP device. The IEC approval ensures that the study adheres to ethical principles, including patient confidentiality, voluntary participation, and informed consent. Specifically, for sensitive information pertaining to medication consumption, the Randomized Response (RR) method was employed to safeguard participant privacy while obtaining accurate data. This ethical framework not only protects the rights of participants but also upholds the integrity and validity of research findings concerning medication adherence and device efficacy among TB patients using STAMP.

Design and function of an automated dispenser, STAMP

**Figure 1:**
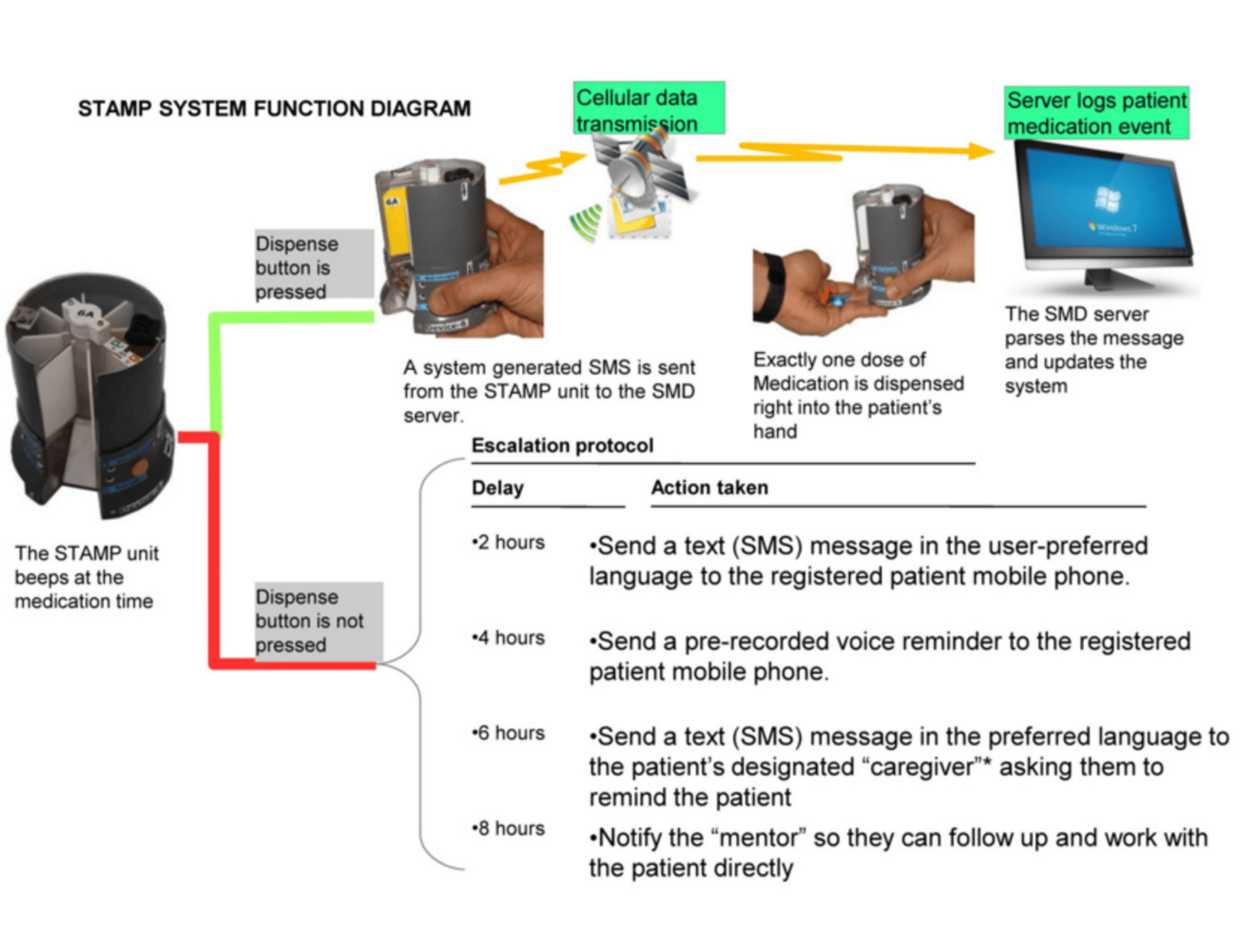
STAMP system function diagram. This diagram illustrates the operational workflow of the STAMP system. It showcases the automated medication dispensing process, real-time data recording via secure SMS protocols, and the escalation of notifications to patients and healthcare providers in cases of missed doses. The diagram underscores STAMP’s role in enhancing medication adherence among tuberculosis patients through innovative technology integration and supportive healthcare interventions. STAMP: Support for Treatment Adherence and Medication Protocols.

**Figure 2:**
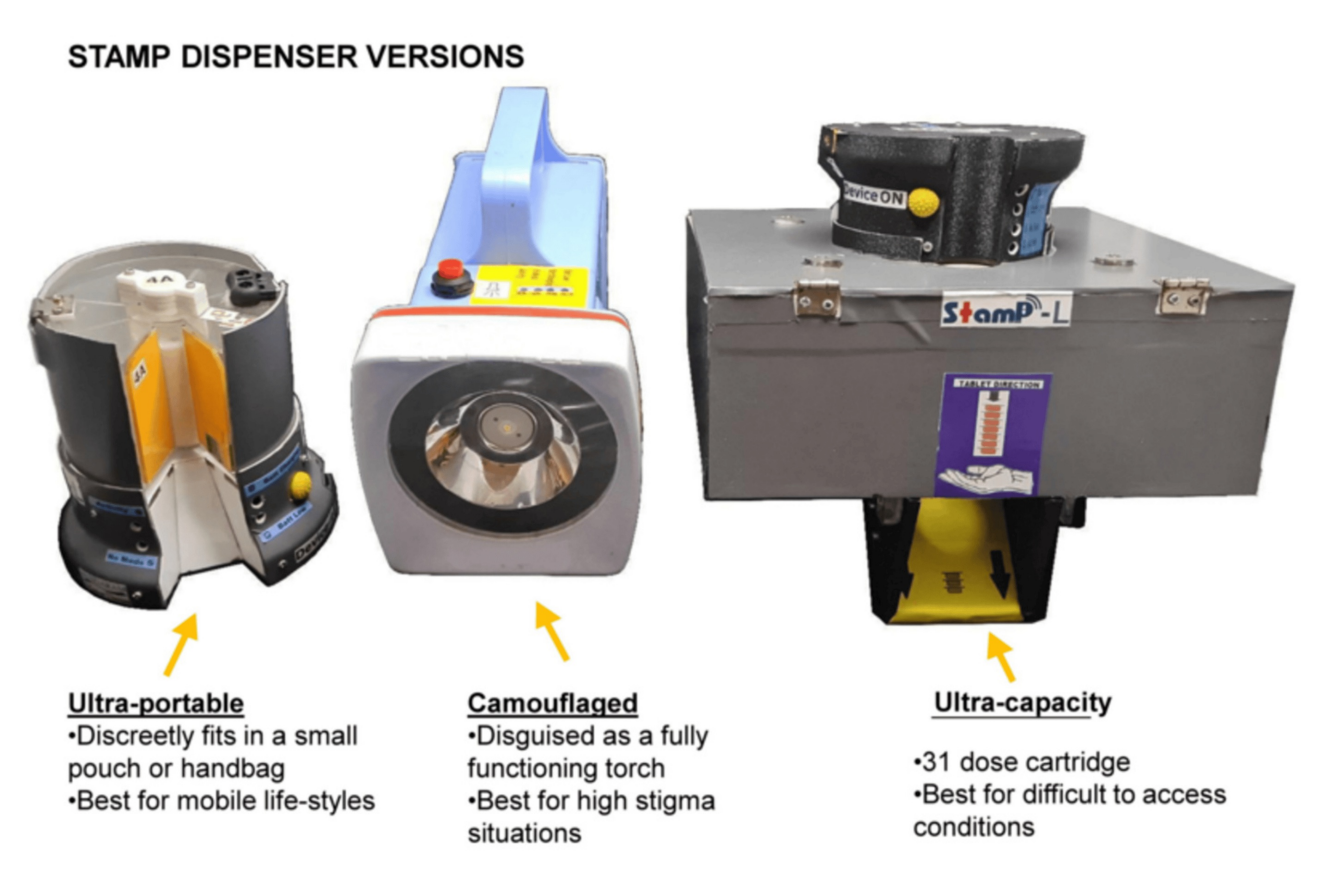
STAMP dispenser versions. This figure displays the three distinct versions of the STAMP dispenser: STAMP UltraPortable, STAMP Camouflaged, and STAMP UltraCapacity. Each version is designed to cater to varying patient needs, offering different levels of portability, privacy, and medication capacity. The diversity in dispenser versions ensures flexibility in deployment across various healthcare settings, supporting tailored management of tuberculosis treatment adherence. STAMP: Support for Treatment Adherence and Medication Protocols.

**Figure 3:**
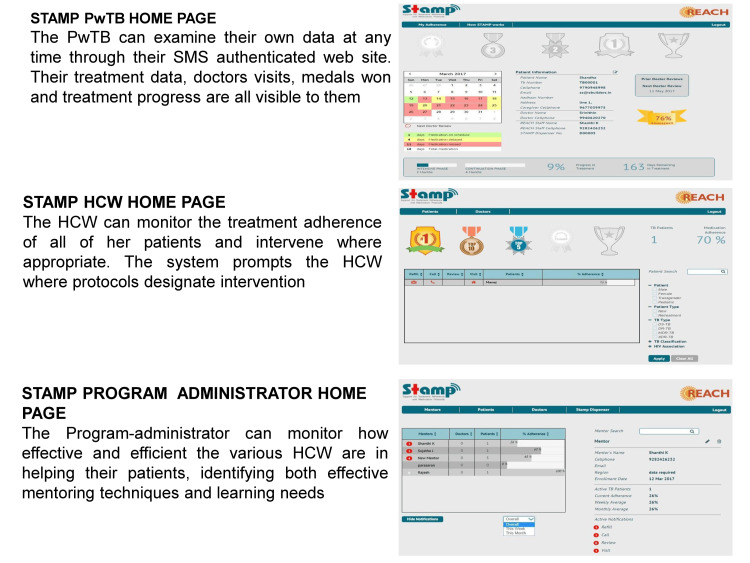
STAMP PwTB home page, STAMP HCW home page, and STAMP program administrator home page. This figure illustrates the user interfaces of the STAMP system, highlighting the home pages for different stakeholders: PwTB, HCW, and Program Administrator. Each interface provides distinct functionalities tailored to the specific roles and responsibilities within tuberculosis treatment management. STAMP: Support for Treatment Adherence and Medication Protocols; PwTB: Patients with Tuberculosis; HCW: Healthcare Workers.

This study focuses on evaluating STAMP, an electronic medication dispenser developed by Sundaram Medical Devices (SMD), within the context of its application across various patient demographics and healthcare environments. STAMP is available in three distinct models: the STAMP UltraPortable, characterized by its compact dimensions of 12 cm in height and 8.6 cm in diameter, designed to accommodate seven dose cartridges for easy mobility; the STAMP Camouflaged, measuring 20 cm in length, 10.8 cm in width, and 16 cm in height, which not only dispenses medications but also functions as a discreet torch to safeguard patient privacy; and the STAMP UltraCapacity, with larger dimensions of 20.6 cm in length, 20.6 cm in width, and 14.4 cm in height, capable of holding 31 doses per cartridge, making it suitable for patients requiring frequent or multiple medication administrations. Each STAMP model operates using a resilient SMS protocol over mobile networks to ensure reliable communication and data transmission. The system is supported by a secure, remote database infrastructure that allows HCWs, program administrators, and patients themselves to access adherence data, appointment schedules, and medication refill reminders. A schematic diagram of the function of STAMP is represented in Figure 1. STAMP is designed to fit varied lifestyles and conditions simply and robustly to help people complete their treatment successfully. Below are the STAMP dispenser versions and cartridge change details in Figure 2. There is a range of STAMP dispensers to meet the variety of needs and situations of a diverse population. Every STAMP unit comes with the dispenser and two cartridges and connects to a common backend. They have a rechargeable battery that runs for one week on an hour’s charge. The ultra-portable and camouflaged versions' cartridges hold a week of medication each, and the ultra-capacity version holds a full 31 doses of medicine. Once the cartridge is empty, the user can replace it with a filled cartridge themselves. During their regular check-ins at the health center, they can turn in the empty cartridges for filled ones. Dashboards for patients with TB (PwTB), HCWs, and program administrators help them monitor medication adherence, treatment milestones, and highlight areas and individuals who might need additional support (Figure 3).

Study design, participants, and data collection

This study utilizes a cross-sectional design to assess TB medication adherence patterns among individuals using the STAMP device. Adherence data were collected over a specified duration, capturing varied age groups and genders. Participants in the study include individuals who have used the STAMP device continuously for at least three months. The data are stratified by age groups ranging from 10 to 15 years to 86 to 90 years, and by gender categories (male, female, transgender). Adherence data were retrieved from electronic records stored on the STAMP server. The collected metrics include on-time adherence, instances of manual overrides by HCWs, delayed medication dispensation, and missed doses. These parameters provide a comprehensive overview of adherence behaviors facilitated by the STAMP device.

Data analysis

Statistical analysis was performed using appropriate software such as SPSS or R. Descriptive statistics, including frequency tables and histograms, were generated to summarize adherence metrics across the study cohort. Inferential statistics, such as Chi-square tests or t-tests, may be employed to identify significant associations or differences in adherence patterns among different age and gender groups. Subgroup analyses will be conducted to explore variations in adherence based on demographic factors and clinical characteristics. Correlation analyses will further investigate potential relationships between adherence metrics and influencing factors such as socioeconomic status, presence of comorbidities, and duration of TB treatment. By systematically analyzing adherence data collected through the STAMP system, this study aims to provide insights into the efficacy of automated medication dispensing and real-time monitoring in enhancing TB treatment adherence. The findings will contribute to optimizing TB management strategies, inform future interventions aimed at improving medication compliance among TB patients globally, and allow more convenient and affordable treatment modes with improved overall outcomes.

Analysis of medication adherence efficacy: a statistical analysis using the RRT

Method 2 introduces a contrasting approach (Plan II), utilizing two contradictory questions associated with differently colored flashcards to address respondent discomfort. Respondents select a flashcard without disclosing its color and respond to a corresponding question about medication consumption. This anonymized method enhances respondent comfort, potentially yielding more truthful responses. The proportion of black and white flashcards is predefined to meet statistical criteria, facilitating accurate estimation of medication non-adherence rates based on survey findings and response probabilities.

In parallel, alongside the device data analysis, a separate survey employing the Randomized Response Technique by Warner SL (1965) has been conducted among a subset of patients [9]. This innovative survey method ensures confidentiality and encourages more accurate reporting of sensitive behaviors, such as medication consumption. By employing such a technique, the study seeks to validate the self-reported adherence data derived from the dashboard analysis. This dual-pronged approach not only enhances the reliability of findings but also provides a more nuanced understanding of patient behaviors related to medication adherence. By integrating these diverse analytical methods, the study aims to provide actionable insights that healthcare providers and mentors can utilize to implement targeted interventions. These interventions are designed to address specific barriers to adherence identified through the analysis, ultimately improving patient compliance and overall health outcomes.

In Method 1, the survey methodology employs two types of questions to address sensitivity and respondent comfort. The survey includes a sensitive question concerning medication adherence, alternated with a non-sensitive question. The strategy aims to influence the proportion of respondents answering the sensitive question based on responses to the non-sensitive one. For instance, respondents are directed to answer the sensitive question based on the nature of their mobile phone number's last digit, promoting varied responses and potentially reducing inhibition. Survey outcomes are analyzed to estimate the proportion of respondents who may not have adhered to prescribed medication, integrating probabilities derived from the survey data.

Method 2 introduces a contrasting approach (Plan II), utilizing two contradictory questions associated with differently colored flashcards to address respondent discomfort. Respondents select a flashcard without disclosing its color and respond to a corresponding question about medication consumption. This anonymized method enhances respondent comfort, potentially yielding more truthful responses. The proportion of black and white flashcards is predefined to meet statistical criteria, facilitating accurate estimation of medication non-adherence rates based on survey findings and response probabilities.

## Results

Overall adherence and nonadherence to the medication from July 2022 to June 2023

**Figure 4:**
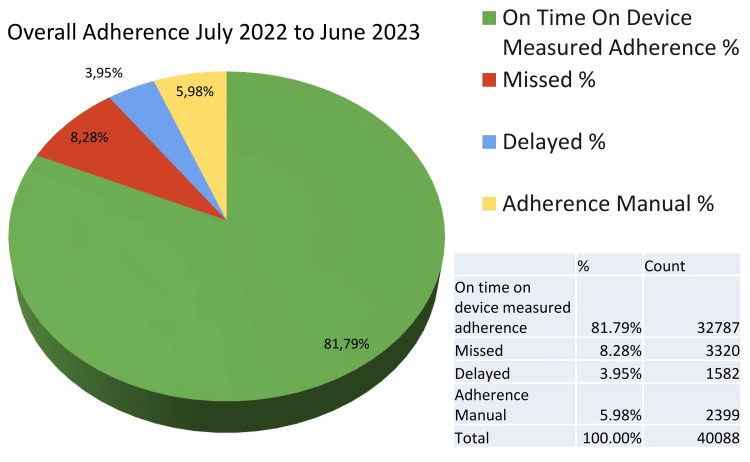
Overall adherence rates according to the STAMP dashboard. STAMP: Support for Treatment Adherence and Medication Protocols.

From July 2022 to June 2023, the STAMP device recorded an On Time On Device Adherence (OTODA) rate of 81.79%, with 8.28% of doses missed and 3.95% consumed late (Figure 4). Manual adherence interventions were implemented in 5.98% of instances. Analysis of non-adherence revealed predominant factors such as out-of-station travel and forgetfulness. Notably, a significant number of patients traveling out of station attempted to mitigate adherence challenges by pre-emptively dispensing medication through multiple presses of the device, either on the day of travel or the preceding day. This proactive behavior underscores patients' efforts in managing their medication despite logistical hurdles associated with travel.

Age and gender-wise adherence patterns in STAMP device usage

**Figure 5:**
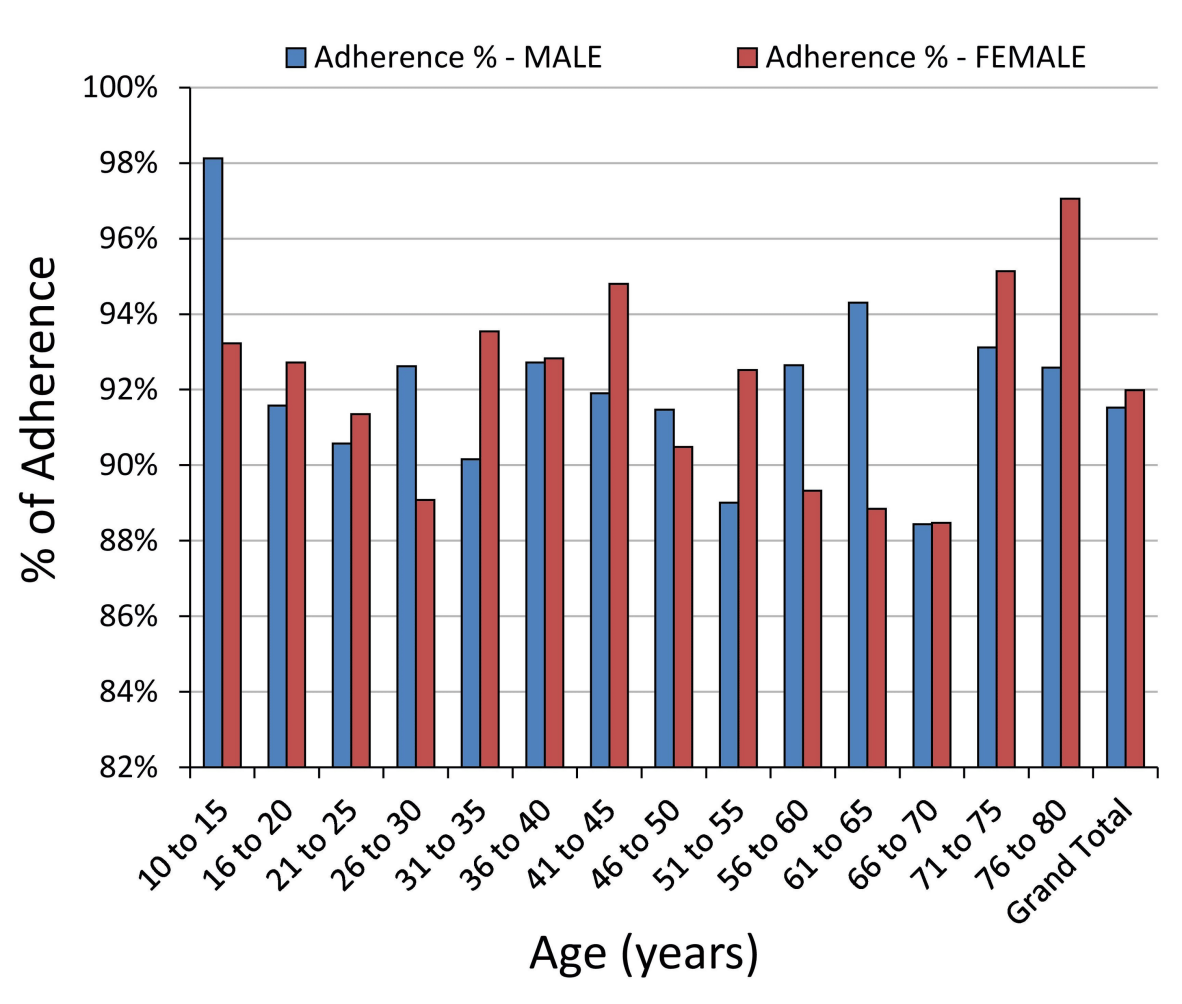
Gender-wise adherence patterns.

In the realm of medical device adherence, insights into demographic patterns are pivotal for understanding patient behaviors and outcomes (Figure 5). This study examines adherence metrics across various age groups and genders, specifically focusing on on-time usage, missed doses, delayed consumption, instances of manual overrides, OTODA, and overall non-adherence rates. A detailed analysis of these parameters, as summarized in Table 1, offers a comprehensive perspective on adherence trends among the study cohort. This data-driven approach provides valuable insights into how demographic factors may influence medication adherence behaviors and outcomes in clinical practice.

**Table 1:** Age-wise adherence analysis - male patients.

Age (Years)	On Time On Device Adherence (OTODA) %	Missed %	Delayed %	Manual %	Adherence %	Non Adherence %	On Time On Device Adherence (OTODA) With Device
10 to 15	88.40%	1.88%	1.57%	8.15%	98.12%	1.88%	89.97%
16 to 20	78.42%	8.42%	5.81%	7.35%	91.58%	8.42%	84.23%
21 to 25	79.96%	9.43%	6.07%	4.55%	90.57%	9.43%	86.02%
26 to 30	84.46%	7.38%	2.60%	5.56%	92.62%	7.38%	87.06%
31 to 35	80.03%	9.84%	4.85%	5.28%	90.16%	9.84%	84.88%
36 to 40	79.58%	7.28%	6.35%	6.79%	92.72%	7.28%	85.93%
41 to 45	83.47%	8.10%	3.21%	5.22%	91.90%	8.10%	86.68%
46 to 50	82.28%	8.53%	4.26%	4.93%	91.47%	8.53%	86.54%
51 to 55	77.21%	10.99%	4.25%	7.55%	89.01%	10.99%	81.47%
56 to 60	84.99%	7.36%	2.30%	5.35%	92.64%	7.36%	87.30%
61 to 65	84.90%	5.69%	3.54%	5.87%	94.31%	5.69%	88.44%
66 to 70	81.69%	11.57%	0.24%	6.51%	88.43%	11.57%	81.93%
71 to 75	85.45%	6.88%	3.44%	4.23%	93.12%	6.88%	88.89%
76 to 80	82.69%	7.42%	1.10%	8.79%	92.58%	7.42%	83.79%
Grand Total	81.40%	8.47%	4.20%	5.93%	91.53%	8.47%	85.60%

The data analysis revealed intriguing patterns in adherence among different age groups and genders. Among individuals aged 10 to 15 years, males demonstrated a higher on-time adherence rate (98%) compared to females (93%). However, females exhibited slightly lower rates of missed instances and delayed usage, resulting in comparable OTODA rates with the device (92% for females vs. 92% for males).

As age increased, overall adherence tended to decrease slightly, with the 66 to 70 age group exhibiting the lowest adherence rates across all categories. Notably, both females (88%) and males (88%) in this age group showed significantly lower OTODA rates compared to other age groups.

Interestingly, the 71-75 and 76-80 age groups highlighted substantial gender differences in adherence behavior. Females demonstrated notably higher adherence rates of 95% and 97%, respectively, whereas males showed slightly lower adherence rates of 93% in both age groups. These findings emphasize the significance of considering age and gender dynamics when developing interventions to enhance adherence among STAMP device users. Such insights could inform personalized healthcare strategies aimed at improving patient outcomes and optimizing treatment.

Age-wise adherence analysis in STAMP device usage

**Figure 6:**
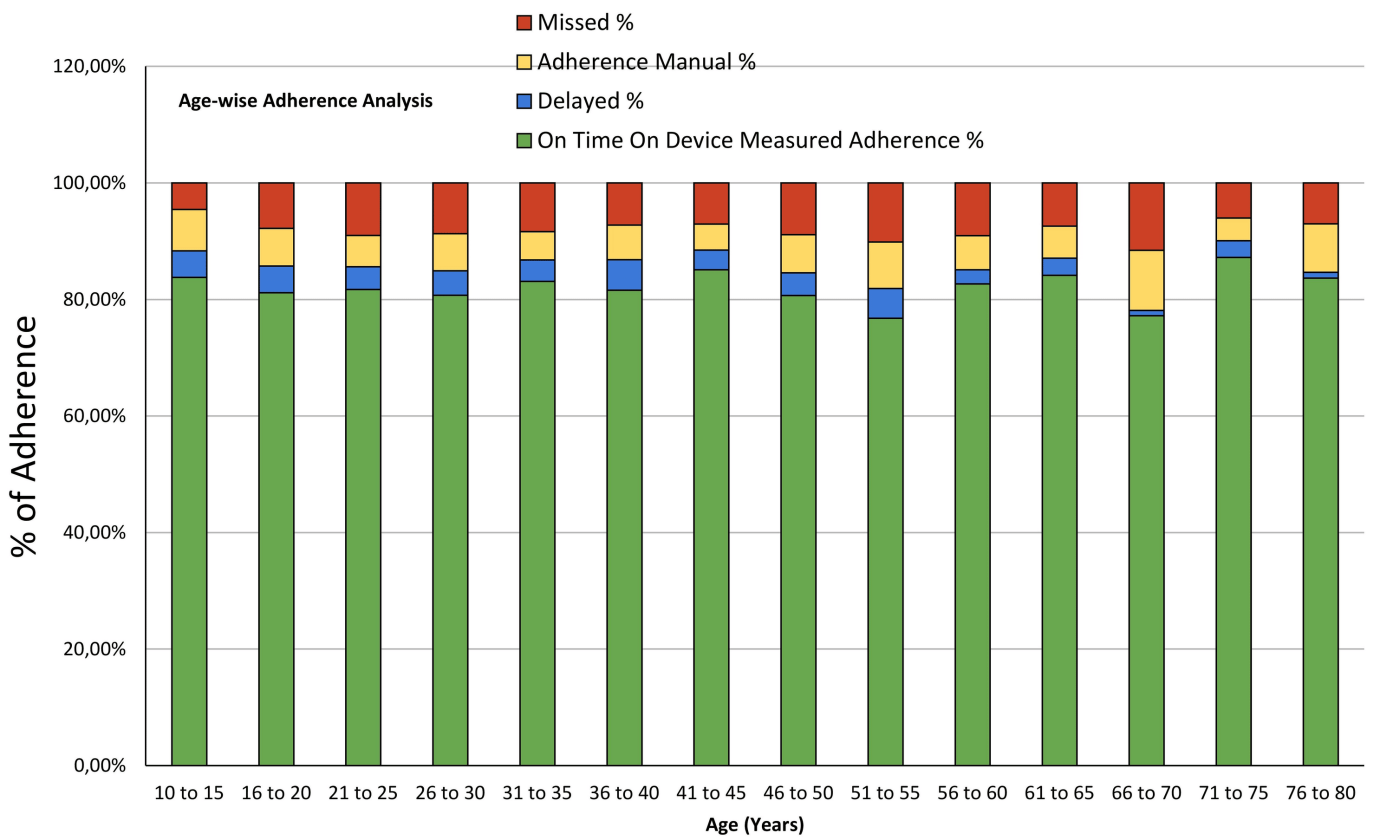
Age-wise adherence analysis.

Understanding adherence patterns across different age groups is essential for enhancing patient outcomes in medical device usage. Figure 6 provides a comprehensive analysis of all the features of the device across distinct age brackets. This detailed examination aims to elucidate how age influences adherence behaviors among users of medical devices, offering insights that can inform targeted interventions and personalized healthcare strategies to improve treatment adherence and efficacy.

The data highlights notable trends in adherence across different age groups. Younger individuals, such as those aged 10 to 15, demonstrate a relatively high OTODA rate of 83.78%. However, as age increases, there is a slight decrease in OTODA, with individuals in the 66-70 age group exhibiting a lower adherence rate of 77.20%.

Analysis of missed instances and delayed usage shows varying patterns. For instance, the 66-70 age group has the highest percentage of missed instances (11.55%) compared to other age brackets, suggesting potential challenges in adherence among older adults. In contrast, the 76 to 80 age group shows a notable decrease in delayed usage (1.01%), indicating better adherence to scheduled device usage within this age range. Overall, the grand total adherence rate stands at 81.79%, indicating substantial adherence across all age groups. However, targeted interventions may be beneficial, especially for older age groups, to further improve adherence rates and enhance patient outcomes in medical device usage. Further research exploring factors influencing adherence within specific age cohorts can provide valuable insights for tailored healthcare strategies.

Age-wise adherence analysis among male patients

**Figure 7:**
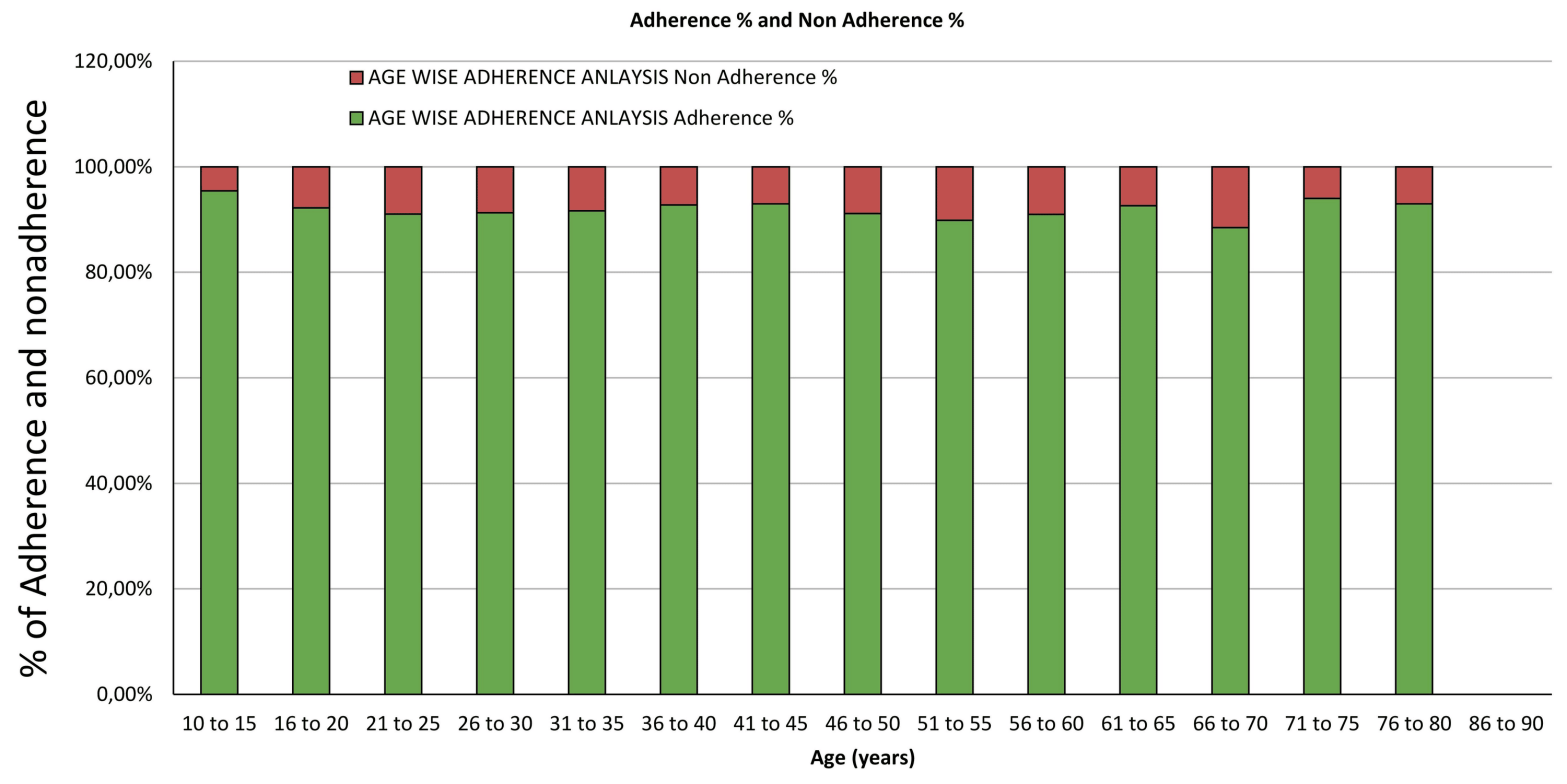
Age-wise adherence analysis - male patients.

The adherence patterns among male patients across different age groups are essential for understanding gender-specific factors influencing medical device usage (Figure 7). Table 1 presents a detailed analysis of OTODA percentages, missed instances, delayed usage, adherence with manual overrides, overall adherence rates, non-adherence percentages, and OTODA with the device among male patients.

Notably, younger males in the 10 to 15 age group exhibit a high OTODA rate of 98.12%, with relatively low rates of missed instances (1.88%). Conversely, older males in the 66 to 70 age group show a lower OTODA rate of 88.43%, with a higher percentage of missed instances (11.57%).

Analysis of manual adherence indicates that younger males tend to have higher rates of adherence with manual overrides compared to older males. For instance, the 10 to 15 age group has an adherence manual percentage of 8.15%, suggesting a higher reliance on manual interventions in this age cohort. Overall, the grand total OTODA rate among male patients stands at 81.40%. These findings underscore the importance of considering age-specific factors when designing interventions to improve medical device adherence among male patients.

Age-wise adherence analysis among female patients

**Figure 8:**
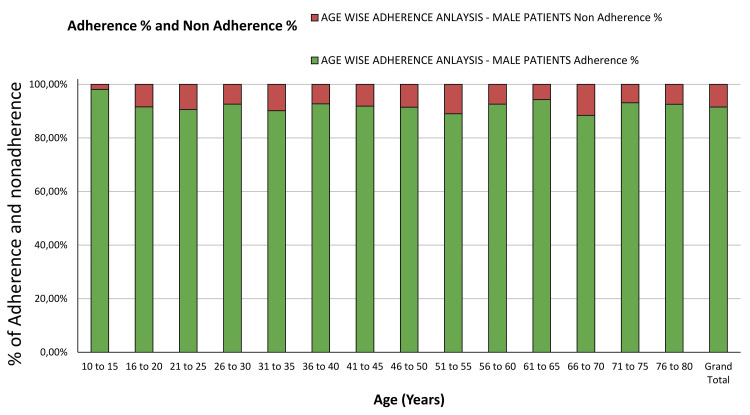
Age-wise adherence analysis - female patients.

Examining adherence patterns among female patients across different age groups is crucial for understanding gender-specific aspects influencing medical device usage (Figure 8). Table 2 provides a comprehensive analysis of the performance of the device among female patients.

**Table 2:** Age-wise adherence analysis - female patients.

Age (Years)	On-time Adherence %	Missed %	Delayed %	Adherence Manual %	Adherence %	Non Adherence %	On Time On Device Adherence (OTODA) With Device
10 to 15	79.95%	6.77%	7.03%	6.25%	93.23%	6.77%	86.98%
16 to 20	83.48%	7.28%	3.54%	5.69%	92.72%	7.28%	87.03%
21 to 25	83.14%	8.65%	2.16%	6.05%	91.35%	8.65%	85.30%
26 to 30	74.57%	10.92%	6.88%	7.63%	89.08%	10.92%	81.45%
31 to 35	87.00%	6.46%	2.33%	4.22%	93.54%	6.46%	89.33%
36 to 40	83.62%	7.17%	4.08%	5.13%	92.83%	7.17%	87.71%
41 to 45	87.88%	5.20%	3.74%	3.17%	94.80%	5.20%	91.62%
46 to 50	77.59%	9.52%	3.15%	9.75%	90.48%	9.52%	80.74%
51 to 55	75.40%	7.48%	7.82%	9.30%	92.52%	7.48%	83.22%
56 to 60	80.44%	10.67%	2.51%	6.38%	89.33%	10.67%	82.95%
61 to 65	82.50%	11.15%	1.54%	4.81%	88.85%	11.15%	84.04%
66 to 70	69.55%	11.52%	2.06%	16.87%	88.48%	11.52%	71.60%
71 to 75	89.58%	4.86%	2.08%	3.47%	95.14%	4.86%	91.67%
76 to 80	94.12%	2.94%	0.00%	2.94%	97.06%	2.94%	94.12%
86 to 90							
Grand Total	82.33%	8.01%	3.61%	6.05%	91.99%	8.01%	85.94%

Younger females in the 10 to 15 age group exhibit an on-time adherence rate of 79.95%, with relatively higher rates of missed instances (6.77%) and delayed usage (7.03%) compared to older age groups. In contrast, older females in the 76 to 80 age group demonstrate a significantly higher on-time adherence rate of 94.12%, with minimal missed instances (2.94%) and no delayed usage. This suggests a trend of improved adherence with age among female patients. Analysis of manual adherence indicates varying reliance on manual overrides across different age groups. Younger females tend to have higher adherence with manual overrides, while older females exhibit lower reliance on manual interventions.

Overall, the grand total OTODA rate among female patients stands at 82.33%. These findings underscore the importance of age-specific considerations when designing interventions to enhance medical device adherence among female patients. Tailored strategies addressing the unique needs and challenges within different age cohorts can contribute to improved adherence rates and better patient outcomes.

Age-wise adherence analysis among transgender patients

Analyzing adherence patterns among transgender patients is crucial for understanding and addressing their unique healthcare needs. Table 3 provides a detailed analysis of OTODA percentages, missed instances, delayed usage, adherence with manual overrides, overall adherence rates, non-adherence percentages, and OTODA with the device among transgender patients.

**Table 3:** Age-wise adherence analysis - transgender patients.

Age (Years)	On Time On Device Adherence (OTODA) %	Missed %	Delayed %	Adherence Manual %	Adherence %	Non Adherence %	On Time On Device Adherence (OTODA) With Device
31 to 35	36.36%	36.36%	9.09%	18.18%	63.64%	36.36%	45.45%
Grand Total	36.36%	36.36%	9.09%	18.18%	63.64%	36.36%	45.45%

The data is analyzed with only one transgender patient, as we had only one transgender participant in the study group. The data highlights challenges in adherence among transgender patients, with an overall OTODA rate of 36.36%. Missed instances and delayed usage are notably high, indicating potential barriers to consistent medical device usage within this demographic. Analysis of manual adherence reveals a reliance on manual overrides, contributing to the overall adherence rate. However, despite these interventions, the non-adherence percentage remains significant at 36.36%. Further research and targeted strategies are needed to address the specific needs and challenges faced by transgender individuals in medical device usage. These findings underscore the importance of considering age and gender dynamics when designing interventions to improve medication adherence. Further research exploring the underlying reasons for these adherence patterns is warranted to inform targeted healthcare interventions. A major limitation of the current study is that self-reported adherence data may be subject to recall bias or social desirability bias.

Enhancing medication adherence efficacy: a statistical analysis using the RRT

While this method provided initial insights into medication adherence rates, it lacked the rigorous verification required to confirm actual consumption. To overcome this limitation, we implemented the Randomized Response Technique, a more reliable and statistically robust approach, to accurately assess medication consumption among TB patients. Given the inherent limitations of self-reported adherence data, it is crucial to precisely determine daily medication consumption using the STAMP device to ascertain true adherence rates. Therefore, this phase of the study involved conducting experiments designed to elicit responses to sensitive questions in two parts, aimed at determining the OTODA rates.

Experiment 1: Eliciting Response to a Sensitive Question

**Figure 9:**
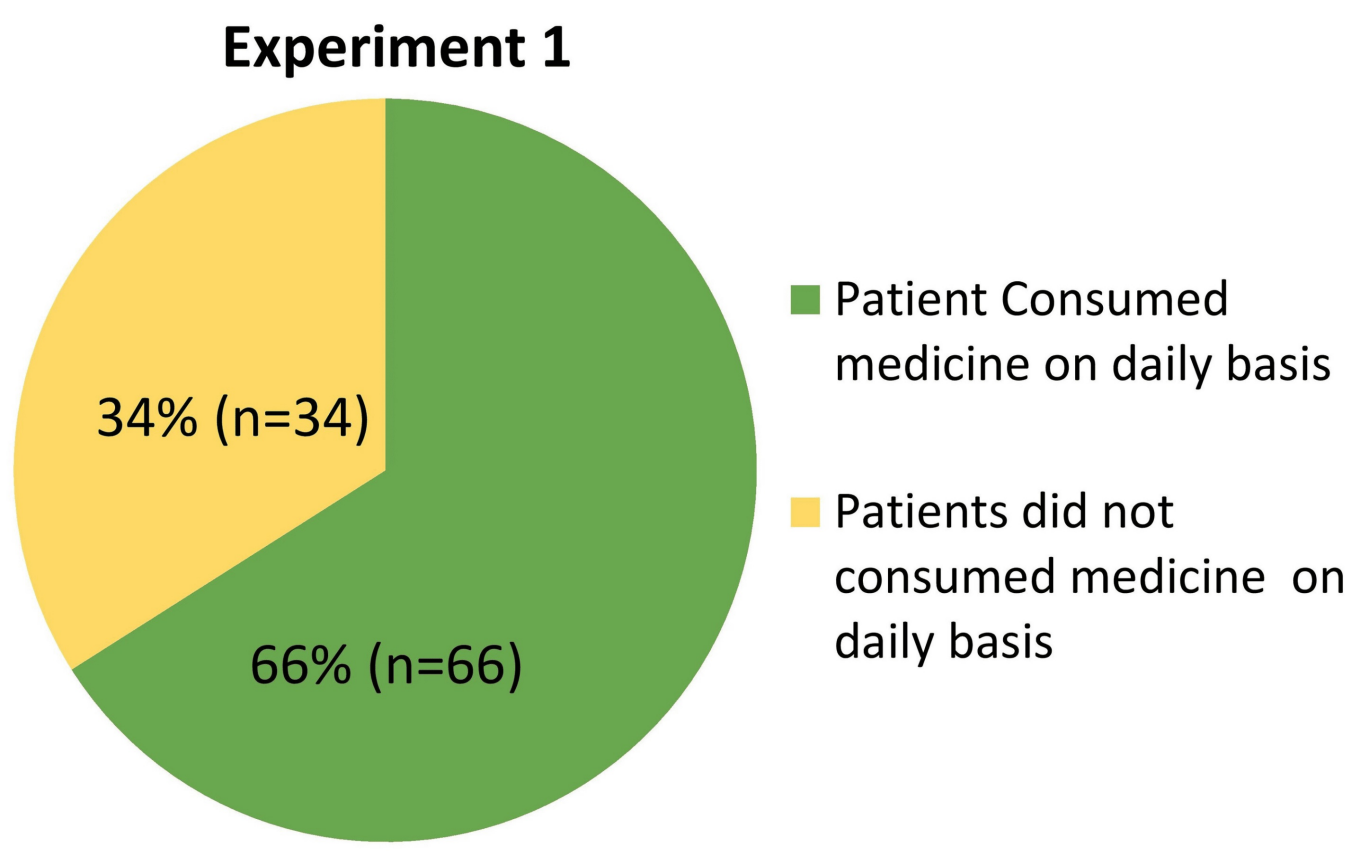
Pie chart representing the distribution of patients' medication consumption after dispensing from the STAMP device. STAMP: Support for Treatment Adherence and Medication Protocols.

In Experiment 1 of the randomized response study, 'Eliciting Response to a Sensitive Question,' we aimed to evaluate medication adherence among STAMP medicine dispenser users using a randomized response technique (Figure 9). Participants were presented with either a non-sensitive or a sensitive question based on the outcome of a coin toss: Question 1 asked about the last digit of their mobile number, while Question 2 directly inquired about daily tablet consumption from the STAMP Dispenser. Out of 100 respondents, 83 indicated adherence to their medication regimen, yielding a compliance rate of 66%. This approach, leveraging probability analysis, effectively minimized response bias and provided a robust quantitative assessment of adherence behaviors. These findings underscore the effectiveness of the STAMP device in promoting regular medication intake and highlight its potential to enhance treatment adherence strategies in clinical settings. Such insights are crucial for optimizing patient care and may guide future interventions aimed at improving therapeutic outcomes for TB patients using automated dispensing technologies.

Experiment 2: Eliciting Response to a Sensitive Question With Altered Probability

**Figure 10:**
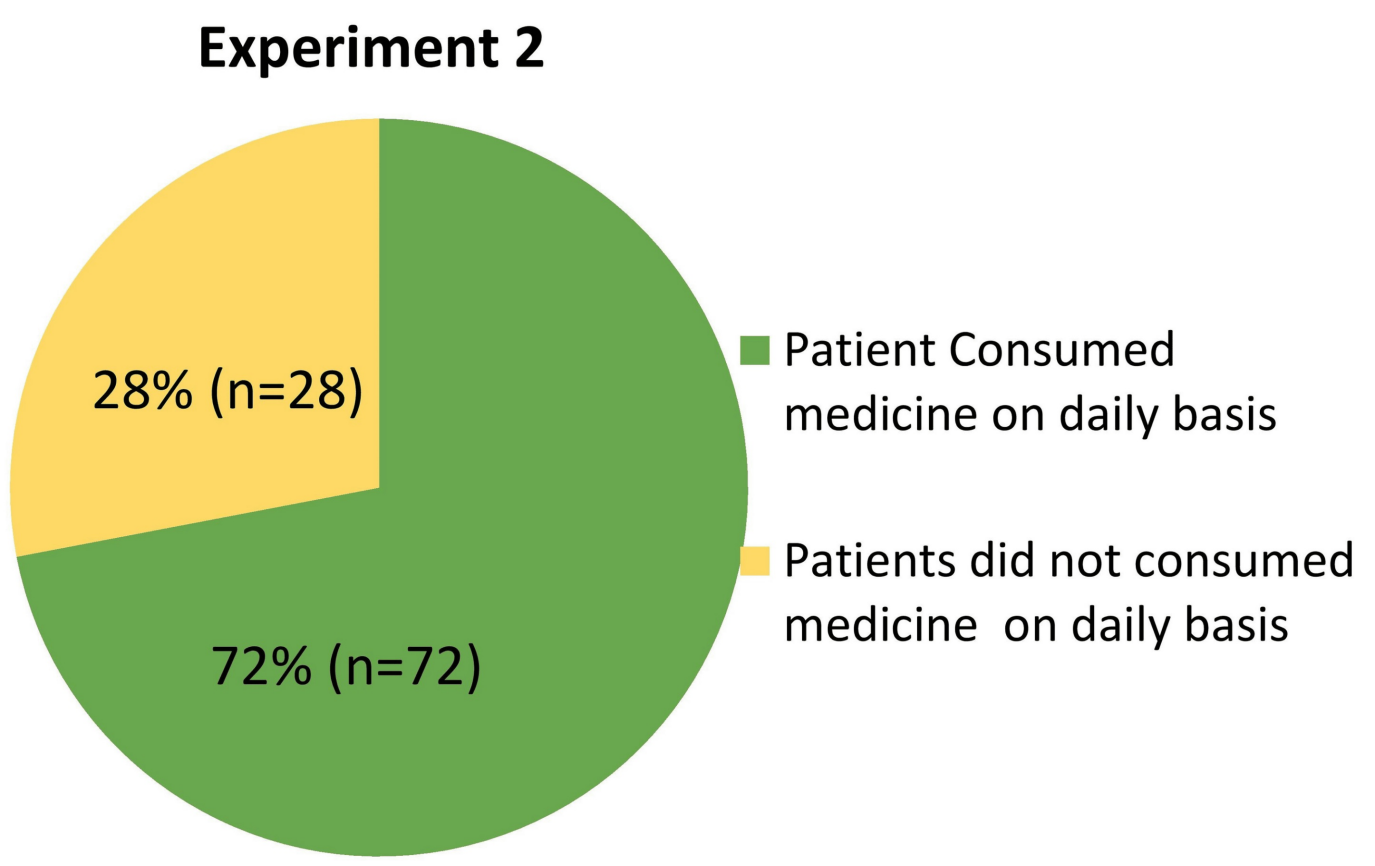
Pie chart showing the distribution of patients’ medication consumption following dispensing from the STAMP device. STAMP: Support for Treatment Adherence and Medication Protocols.

The second experiment of our phase 2 study, titled 'Eliciting Response to a Sensitive Question with Altered Probability,' utilized a novel approach involving flash cards to investigate tablet consumption among STAMP medicine dispenser users (Figure 10). This methodology aimed to enhance respondent anonymity and encourage truthful responses by varying the probability of card selection and question presentation. Participants were presented with flash cards featuring a uniform pattern on one side, with 80% of cards black-colored and 20% red-colored on the other side. They randomly chose one card without revealing their selection. Depending on the card's color: Black cards prompted participants to respond to Question 1: 'Have you consumed all the tablets dispensed by the STAMP medicine dispenser on a daily basis?' with answer options 'Yes' or 'No'. Red cards prompted participants to respond to Question 2: 'Have you not consumed all the tablets dispensed by the STAMP medicine dispenser on a daily basis?' also with answer options 'Yes' or 'No'.

Participants were not required to disclose which question they answered or which card they selected, ensuring confidentiality and potentially facilitating more candid responses. A total of 100 patients participated in the study. The responses were analyzed using the formula YA=p×EP+(1−p)×(1−EP), where YA represents the calculated compliance (tablet consumption) rate, p denotes the probability of selecting a black card (0.80), and EP represents the probability of responding affirmatively (0.72). The analysis revealed a compliance rate of 72% among participants, indicating that 72 out of every 100 patients consumed the tablets dispensed by the STAMP medicine dispenser on a daily basis. The non-compliance rate was calculated at 28%. These results provide quantitative insights into medication adherence behaviors among STAMP device users under conditions designed to minimize respondent bias. By employing a randomized selection of questions linked to anonymous responses via flash cards, this experiment aimed to mitigate social desirability bias and improve the accuracy of adherence assessments. The high compliance rate observed suggests that the altered probability approach effectively encouraged more truthful reporting compared to traditional methods reliant solely on self-reporting or direct questioning.

The findings underscore the potential of innovative methodologies, such as altered probability techniques using flash cards, to enhance the reliability of data collected on medication adherence. Future research could further explore variations in probability settings and expand the application of similar techniques to different patient populations and healthcare contexts, ultimately advancing precision medicine approaches for chronic disease management [10-13].

## Discussion

The STAMP system represents a significant advancement in the field of medical device technology, particularly in its application to TB treatment adherence. As a multifunctional electronic medication dispenser, STAMP addresses several critical challenges in patient compliance, a cornerstone of successful TB treatment. This discussion delves into the design elements, functionality, and implications of the STAMP system in promoting adherence across diverse patient populations.

One of the key strengths of the STAMP system lies in its user-centered design, which tailors the device to meet the needs of different patient demographics. The three distinct models, STAMP UltraPortable, STAMP Camouflaged, and STAMP UltraCapacity, each serve specific purposes, from high mobility and discretion to large capacity for frequent medication administration. The UltraPortable model is particularly beneficial for younger, active patients or those who are frequently on the move, offering them the flexibility to maintain adherence without being burdened by a large device. The Camouflaged model, which doubles as a torch, provides an added layer of privacy and security, catering to patients in environments where discretion is paramount. Lastly, the UltraCapacity model supports patients who require more frequent dosing, making it ideal for those with complex medication regimens. The emphasis on portability, privacy, and capacity reflects a deep understanding of the challenges patients face in adhering to TB treatment, which often requires prolonged medication courses under difficult circumstances. By designing models that integrate seamlessly into varied lifestyles, STAMP enhances the likelihood of consistent medication use [10-13].

STAMP's integration of a robust SMS protocol over mobile networks for real-time monitoring is another notable innovation. This feature ensures that adherence data is reliably transmitted to a secure, remote database, where HCWs and program administrators can monitor patient compliance. The use of SMS technology, which is accessible even in low-resource settings, enhances the system’s utility across different geographical regions, particularly in areas where internet connectivity may be unreliable [14].

The real-time data transmission also allows for immediate interventions in cases of missed doses, a critical function in TB treatment where non-adherence can lead to drug resistance and treatment failure. By alerting patients and HCWs of missed doses, the STAMP system facilitates timely reminders and support, significantly reducing the risk of non-adherence [15]. The study’s findings indicate a substantial overall adherence rate of 81.79%, a figure that underscores the effectiveness of the STAMP system in promoting consistent medication use. However, the study also reveals that certain demographics, particularly older patients, exhibit lower adherence rates. This suggests that while STAMP is effective, further customization or additional support mechanisms may be needed to address the unique challenges faced by older patients.

The variation in adherence across different age groups and genders highlights the complexity of adherence behaviors. For instance, younger males demonstrated higher on-time adherence compared to females in the same age group, while older females outperformed their male counterparts. These findings suggest that adherence strategies need to be tailored not only to the patient’s clinical needs but also to their demographic characteristics [16].

Despite its many strengths, the STAMP system faces several challenges. For one, the reliance on self-reported data, even when supplemented with real-time monitoring, may introduce biases such as recall bias or social desirability bias. The study’s use of the RRT to assess the accuracy of self-reported adherence is a commendable effort to mitigate these biases, but it also highlights the inherent difficulty in obtaining reliable adherence data. Moreover, the system’s reliance on mobile network coverage, while generally robust, may still pose challenges in extremely remote or underserved areas. Although SMS is a low-bandwidth solution, ensuring that all patients, especially those in rural areas, have consistent access to the necessary mobile networks is crucial for the system’s success.

Another limitation is the need for ongoing patient education and support. While the STAMP system provides the tools for adherence, patients must still be motivated to use the device correctly. This is particularly important in older populations, who may struggle with the technology or forget to charge the device, as suggested by the lower adherence rates observed in older age groups. The STAMP system’s success in enhancing TB treatment adherence has significant implications for global health, particularly in low- and middle-income countries where TB remains a major public health challenge. The scalability of the STAMP system, supported by its modular design and use of widely available technology, makes it a promising tool for large-scale deployment in TB control programs.

Future research should focus on refining the system to address the challenges identified in this study. For instance, developing more intuitive interfaces or automated charging reminders could help improve adherence among older patients. Additionally, exploring alternative data transmission methods, such as satellite communication for extremely remote areas, could further extend the system’s reach [17]. The study also underscores the importance of integrating behavioral health strategies into adherence programs. Understanding the psychological and social factors that influence adherence is key to developing more effective interventions. The use of tools like the RRT to gain insights into patient behavior can inform the design of these interventions, making them more effective and culturally appropriate.

From July 2022 to June 2023, the STAMP device demonstrated a solid OTODA rate of 81.79%, highlighting consistent medication adherence. However, challenges persisted, with 8.28% of doses missed and 3.95% delayed, necessitating 5.98% manual interventions. Analysis revealed that out-of-station travel and forgetfulness were the primary causes of non-adherence. Interestingly, some patients attempted to counteract travel-related adherence issues by pre-dispensing multiple doses, indicating a proactive approach to maintaining their medication routine despite logistical difficulties. This data underscores the importance of addressing specific factors that lead to non-adherence, particularly in patients with travel commitments. By understanding these behaviors, healthcare providers can develop targeted strategies to further enhance adherence rates. The results emphasize the value of supportive measures, such as reminders and travel-friendly solutions, to mitigate non-adherence and ensure optimal therapeutic outcomes. This analysis provides a comprehensive understanding of adherence patterns and offers actionable insights for improving patient management and adherence to prescribed regimens [10-13].

Overall, the STAMP system represents a significant advancement in the field of medical adherence technology. Its thoughtful design, technological integration, and focus on real-time monitoring make it a powerful tool for improving TB treatment adherence. While challenges remain, particularly in reaching the most vulnerable populations and ensuring consistent use of the device, the STAMP system’s potential to transform TB treatment adherence on a global scale is undeniable. Future efforts should focus on addressing these challenges and leveraging the system’s strengths to maximize its impact on patient outcomes.

## Conclusions

The STAMP device, an advanced AMD with real-time monitoring capabilities, shows great potential for improving medication adherence in TB patients. The study's findings reveal that the automated nature of the STAMP device, along with the supportive function of healthcare mentors, resulted in an impressive overall OTODA rate of 81.79%. This finding highlights the device's usefulness in reducing common hurdles to adherence, such as forgetfulness and complex drug schedules. When compared to previous technologies such as 99DOTS, MERM Box, and Video Observed Therapy (VOT), STAMP provides significant advantages. While 99DOTS relies significantly on patient input for adherence tracking, and MERM Box sends reminders but lacks real-time intervention capabilities, STAMP fills these gaps by automating the dispensing procedure and including real-time adherence monitoring. The constant oversight provided by the web-linked dashboard enables healthcare personnel to intervene quickly, providing personalized support, which is a considerable improvement over older techniques. Furthermore, the study emphasizes the need to consider demographics such as age and gender when creating treatments. For example, older patients had poorer adherence rates, underscoring the need for tailored interventions to enhance compliance in this population.

Furthermore, the innovative RRT used in the study improved adherence evaluation by decreasing biases associated with self-reported data. Experiment 1, which used coin flip probability and two questions to test adherence, yielded an overall compliance rate of 66%. In contrast, Experiment 2 used altered probability flashcards to increase respondent anonymity, resulting in a 72% pill consumption compliance rate. These discrepancies highlight the role of survey methodology in encouraging more accurate responses about medication adherence behaviors. Additionally, the overall OTODA rate from July 2022 to June 2023 was 81.8%, with consumption rates of 80.7% and 88.0% in Experiments 1 and 2, respectively. These numbers illustrate the stability of adherence trends across several survey methodologies, as well as the device's efficiency in enhancing user adherence. In summary, the STAMP device offers a significant improvement in TB treatment adherence, providing a holistic solution that addresses the limitations of existing technologies. To maximize the device's impact on global TB management strategies, further research should focus on optimizing its functionality, investigating its scalability, and integrating it into a variety of healthcare settings. Furthermore, ongoing research into innovative survey methodology, as illustrated in Experiment 2, is critical for improving data accuracy and shaping personalized healthcare approaches to enhance patient adherence and treatment efficacy.

## Figures and Tables

**Figure 11:**
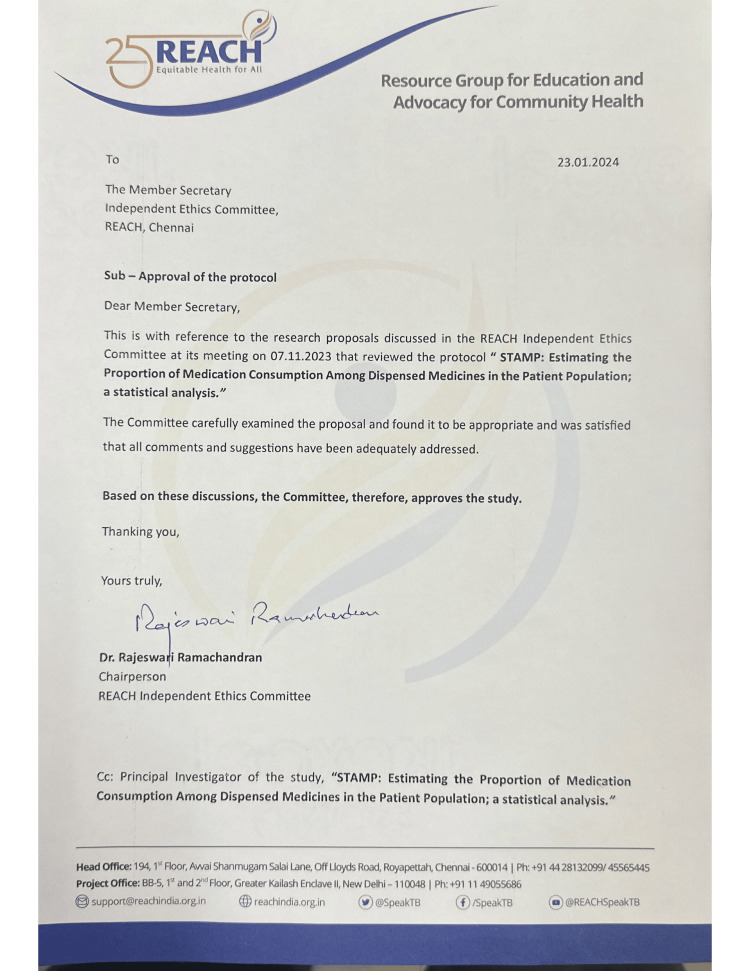
Ethical approval document.

## Appendices

Appendix 1 
